# Supplementary material for: Offline events and online hate
Source: PLoS One. 2023 Jan 25;18(1):e0278511. doi: 10.1371/journal.pone.0278511 (PMC9876356; doi:10.1371/journal.pone.0278511)
Supplement: S1 File — (PDF) [file pone.0278511.s001.pdf]

Offline Events and Online Hate
Supplementary Information

December 1, 2022

1 Data Collection

This section describes how we compiled the list of online communities we tracked and collected data from. To begin with, we focus on online hate communities in order to be able to both construct a training data set that contains a large and diverse set of examples of hate speech, and to, subsequently, construct a larger data set with sufficient observations of hate speech to allow us to analyze changes over time. To perform our analyses we needed a data set that contained many examples of each of the 7 types of hate speech we studied. It was also important for these examples to reflect a wide range of word choices for each of these 7 types of hate speech. In other words, we needed a data set that contained a large and diverse set of examples of hate speech. Especially on moderated platforms, hate speech has become infrequent on all but a few communities. Even on fringe platforms, users who espouse hateful views tend to congregate on relatively few communities, perhaps because they seek to communicate with others who share their views and likely also because of the potential for moderation elsewhere (e.g., 4Chan, as explained below). It would therefore not have been practical for us to collect a sufficient set of observations that contained hate speech at the scale of this project without first focusing on the communities in which it is consistently used. Users on different platforms tend to use language differently. This manifests also in their use of hate speech. This is why we made sure to collect large numbers of posts across the platforms. The only platform in which this proved difficult was Instagram. We spent significant effort searching for other hate communities on Instagram and were not able to find any additional ones, likely because the platform has been effective in removing hate speech (at least in posts).

The method used was snowball sampling. We identified candidate communities by searching for hate-related terms in platform search engines. We searched for the terms found in the Anti-Defamation League Hate Symbols Database (`https://www.adl.org/hate-symbols`) and the names of hate groups tracked by the Southern Poverty Law Center (`https://www.splcenter.org/issues/hate-and-extremism`). Having identified these candidate commu-

nities, we then parsed the URLs they posted to other online communities, and included the linked-to communities as additional candidate communities. Next, we analyzed the set of candidate communities to determine which met our definition of hate communities. We included a community in our data as a hate community if 2 (or more) of the 20 most recent posts on the community included hate speech. For purposes of this step in the process, we defined hate speech as either (a) content that would fall under the provisions of the United States Code regarding hate crimes or hate speech according to Department of Justice guidelines; or (b) content that supports or promotes Fascist ideologies or regime types (i.e., extreme nationalism and/or racial identitarianism), as defined by [1]. The determination of whether an online community is a hate community was made manually using these criteria. We repeated this process several times during the study period to identify new online hate communities. The study includes 1150 online hate communities. Table 1 lists the platforms included in this study, along with the sums of posts and individual communities on each platform.

Perhaps the most well-known of the communities in our data is the Politically Incorrect forum on 4Chan. This forum is perhaps the most important and influential hate community on the Internet, and most likely its most active. In fact, the single forum yielded the bulk of our data, as shown in Table 1. Politically Incorrect, or "/pol/" is known as both the central gathering place for online hate and extremism and as a forum in which users believe they can speak freely without fear of moderation. The forum was created in order to centralize and contain the hate content on 4Chan, so that it can be kept off of other forums on the site [2-3]. 4Chan users often make explicit reference to the notion that they could not say what they say on /pol/ elsewhere on 4Chan without fear of content moderation. In terms of 4Chan, therefore, this is why we included only 1 community.

Table 1: Posts and Communities by Platform

Platform	Posts	Communities
4Chan	57,032,220	1
Facebook	813,051	315
Gab	428,001	330
Instagram	5,468	58
Telegram	839,211	274
VKontakte	59,562	172

2 Training Data

Four annotators coded the training data based on the following definitions. Two of the annotators were subject-matter-expert members of our team with multiple years of experience studying online hate speech. The other two coders received 8 hours of training from our subject-matter experts, including detailed explanations of each hate speech type and extensive sets of examples and practice posts to code. In addition, we conducted the inter-coder reliability analysis described below. A post was deemed to contain hate speech if it advocated or practiced hatred, hostility, or violence toward members of a race, ethnicity, nation, religion, gender, gender identity, sexual orientation, or immigrant group. Posts were coded as containing religion hate if they advocated or practiced hatred, hostility, or violence towards a given religious group and/or advocated supremacy of a religious group over another. Posts were coded as containing race hate if they advocated or practiced hatred, hostility, or violence toward a race and/or advocated racial superiority/supremacy. Posts were coded as containing gender hate if they advocated or practiced hatred, hostility, or violence toward a gender. Posts were coded as containing gender identity / sexual orientation (GI/SO) hate if they advocated or practiced hatred, hostility, or violence toward a gender identity and/or sexual orientation. Posts were coded as containing immigration hate if they advocated or practiced hatred, hostility, or violence toward a group based on its immigration status. Posts were coded as containing ethnicity/identitarian/nationalism (E/I/N) hate if they (1) advocated or practiced hatred, hostility, or violence toward an ethnic group; (2) advocated or

practiced an ideology asserting the exclusive right (i.e., to the exclusion of others) of people of a certain identity or nation to a certain culture or territory; or (3) advocated or practiced ethnic, identitarian, or nationalist superiority. Posts were coded as containing anti-semitism hate if they advocated or practiced hatred, hostility, or violence toward Jews, Judaism, or Jewish institutions and/or religious facilities.

Figure 1 provides descriptive information regarding the full training data set of 31,323 posts. Panel A shows how many posts contained each type of hate. The most common type of hate in the training data is race, as it is in the machine-coded data reported in the main manuscript, followed by E/I/N, GI/SO, and antisemitism in similar quantities. Panel B shows the number of posts containing different amounts of hate speech types. As with the machine-coded data, most posts did not contain hate. Of the posts that did contain hate, they contained an average of 1.33 types of hate speech, a figure similar to that for the machine-coded data. As panel C demonstrates, the majority of the training data were 4Chan posts, which is also the case for the larger data set. The training data include posts from all six platforms.

We conducted the following inter-coder reliability analysis. Coder A re-coded a total of 1000 randomly selected posts initially coded by Coders B, C, and D. Coder B re-coded a total of 1000 randomly selected posts initially coded by Coders A, C, and D. Our analysis of these results is shown in Tables 2 and 3. Agreement between coders is high, but this is likely driven in part by a large number of posts that clearly do not contain a given type of hate speech. We therefore focus on the commonly used statistic of Cohen's Kappa, which measures inter-coder reliability while taking into account the probability they would agree by chance. With respect to the re-coding by Coder A, Cohen's Kappa ranges from 0.84 to 0.95, depending on the type of hate speech. With respect to the re-coding by Coder B, Cohen's Kappa ranges from 0.81 to 0.96. All of these values are above the standards used by the literature, indicating that the coding of the training data set is highly reliable. Posts with respect to which there was disagreement between coders were reviewed by the

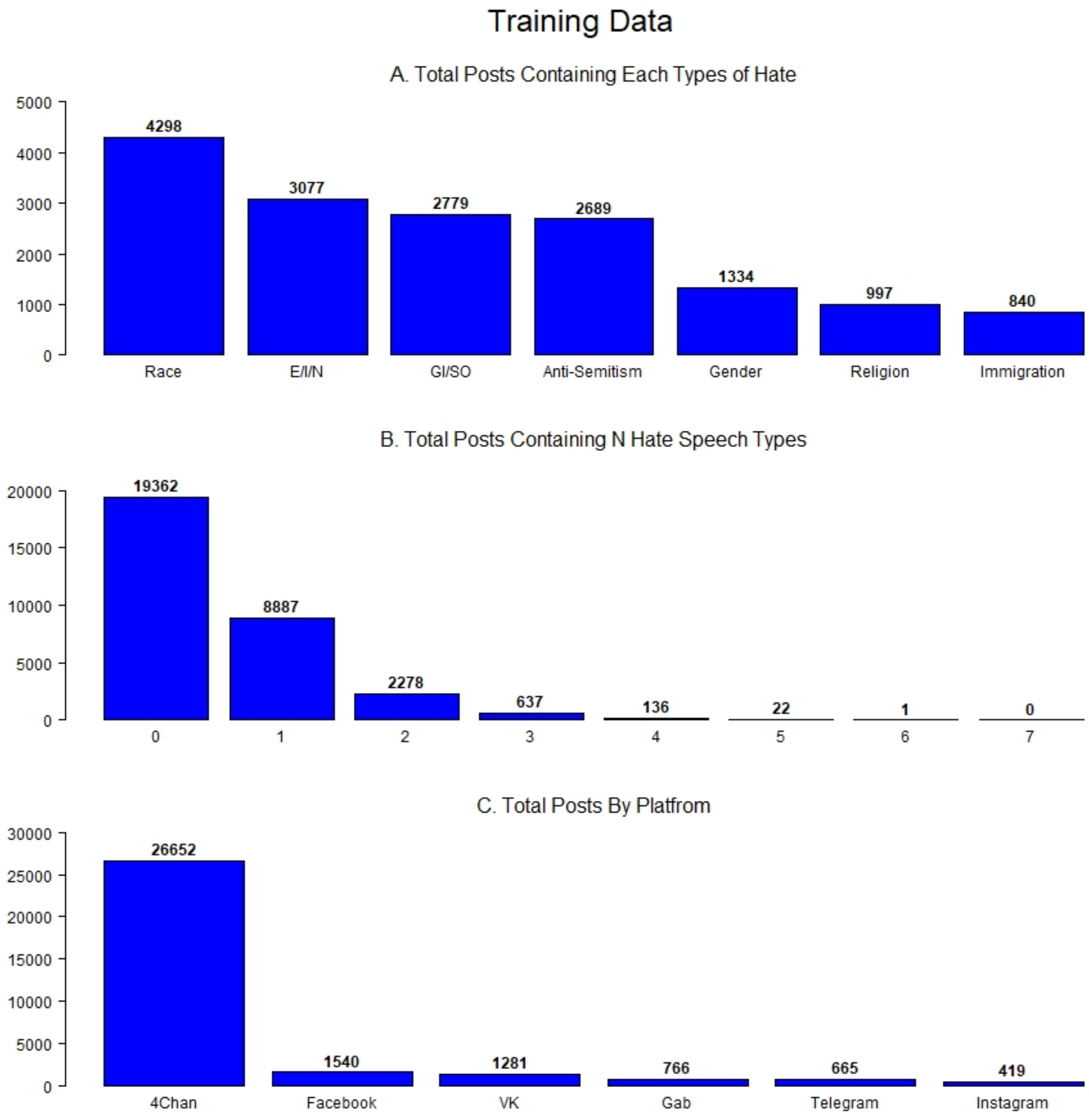

Figure 1: Training Data Summary

subject-matter experts on our team and given a final coding based on their consensus.

	Agreement	Expected Agmnt.	Cohen's Kappa	SE	Z	p>Z
**Religion**	99.70%	97.73%	0.8681	0.0316	27.48	0.0000
**Race**	98.40%	79.95%	0.9202	0.0316	29.12	0.0000
**Gender**	99.50%	94.84%	0.9031	0.0316	28.61	0.0000
**GI/SO**	98.90%	92.96%	0.8437	0.0314	26.90	0.0000
**Immigration**	99.80%	95.31%	0.9573	0.0316	30.30	0.0000
**E/I/N**	99.00%	89.43%	0.9054	0.0316	28.64	0.0000
**Anti-Semitism**	99.10%	83.37%	0.9459	0.0316	29.91	0.0000

Table 2: Inter-Coder Reliability - Coder A

	Agreement	Expected Agmnt.	Cohen's Kappa	SE	Z	p>Z
**Religion**	99.80%	94.56%	0.9633	0.0316	30.48	0.0000
**Race**	99.20%	73.39%	0.9699	0.0316	30.68	0.0000
**Gender**	98.80%	92.32%	0.8438	0.0315	26.83	0.0000
**GI/SO**	98.70%	81.13%	0.9311	0.0316	29.45	0.0000
**Immigration**	99.90%	97.92%	0.9519	0.0316	30.14	0.0000
**E/I/N**	97.80%	88.01%	0.8166	0.0313	26.06	0.0000
**Anti-Semitism**	98.90%	87.41%	0.9126	0.0316	28.87	0.0000

Table 3: Inter-Coder Reliability - Coder B

3 Machine Learning Methods and Model Selection

To split the training data, we used an implementation of Algorithm 1 described by [4] to construct a data set that is representative of the relative distribution of each classification as it appears in the full training data set. We used the following parameters for the stratification algorithm: $k = 2$ (a train and test set), $r_1 = 0.8$, and $r_2 = 0.2$. (In an analogous task in which each class is mutually exclusive, this would be the commonly used 80-20 train-test split.) This yields 26,354 posts used to train the machine and 4,969 used to test performance.

We tested several well-established machine learning model architectures. For basic text representation, we used the term-frequency-inverse document frequency (TF-IDF) algorithm, which weights words according to their prevalence both in individual posts and across the entire text corpus. The basic classifiers we tested are a Naïve-Bayes classifier (NB), a logistic regression classifier (LogReg), and a support vector machine (SVM). A Naïve-Bayes classifier makes decisions based on Bayes' theorem, thereby imposing an (often incorrect) assumption of independence among features it is given. A logistic regression classifier attempts to fit a logistic curve to a given set of data and makes future predictions based on this fitted curve. A support vector machine attempts to learn high-dimensional boundaries between classes, allowing it to make predictions based on where future data lies in relation to these boundaries. We also tested several more advanced architectures. We used a feed-forward neural network (NN), which learns thousands to millions of parameters in a setup that crudely simulates the pattern of neurons in a human brain. Our neural network architecture is simple, using only one dropout layer (with a dropout rate of 0.1), the output of which is used for 7 separate densely connected layers (one for each type of hate speech) with logistic activation functions. We also tested a more advanced convolutional neural network (CNN). This is much like the simple neural network, but before the final densely connected layers, it includes three convolutional layers and a dropout layer. Our CNN also used BERT for text representation. Lastly, we tested a bi-directional long short-term memory (Bi-LSTM) model architecture, a type of recurrent neural network. This architecture functions differently

from feed-forward neural network by attempting to learn relationships in sequential data; in natural language processing, this means sequences of words. While this architecture is theoretically best-suited to text data, previous work [5] has shown that it does not always better than feed-forward approaches, especially on short texts.

While the TF-IDF text representation method is efficient, its key downside is that it does not take into account word context. In the study of hate speech, word context can be particularly important in determining meaning. Notably, this pitfall with respect to TF-IDF is harmful to a neural network, which is suited for learning underlying data patterns that are not necessarily present in the context-less TF-IDF vectors. Recent advancements in natural language processing have shown that context-based transformer models can improve performance. We therefore employ a pretrained bidirectional encoder representation from transformers (BERT) model as an alternative method for text representation. This method takes into account word context with parameters from pre-training on a large, generic corpus of text. We use the Hugging Face Transformers Python library's Tensorflow interface [6] to construct our BERT models. During training, we use a categorical cross-entropy loss function with an Adam optimizer, and the BERT weights are frozen. We tested BERT models with the SVM, CNN, and NN classifiers.

For each of these 8 model architectures (except Bi-LSTM), we train 7 distinct models (one for each hate speech type), each of which separately learns from the same text data. Each of these models performs binary classification for one of the 7 types of hate speech. In the case of Bi-LSTM, we train a single model which is capable of producing seven separate binary predictions for each hate type. This choice is due to both the computational intensity of the Bi-LSTM architecture, as well as its commonly-accepted use in general practice. We evaluate the performance of the 8 model architectures with respect to each of the 7 types of hate speech. Tables 4 through 10 compare the performance of the 8 alternative model architectures for each type of hate speech. Accuracy refers to the share of test posts the model correctly classified. Because the training data set is imbalanced between posts that

contain hate speech and those that do not, we also separately calculate accuracy for set of the posts that contain hate. Precision and recall are often employed as more insightful alternatives to simple accuracy. Precision measures the extent to which items selected by the model are relevant; it is the fraction of true positives out of the sum of true positives and false positives. Recall measures how many of the relevant items are selected by the model; it is the fraction of true positives out of the sum of true positives and false negatives. The F1 score is the harmonic mean of precision and recall: $2(\frac{P*R}{P+R})$. The harmonic mean is used because it penalizes the algorithm for extreme values more than a simple mean would.

As Tables 4 through 10 demonstrate, the basic neural network classifier with BERT embeddings (BERT-NN) overall performs better across metrics and types of hate speech. We therefore select this model architecture to classify the remaining posts in our data set. All of the results in the main manuscript and the remainder of this SI are based on this model architecture.

	**Accuracy**	**Accuracy (hate only)**	**Precision**	**Recall**	**F1**
**TFIDF-NN**	0.970	0.916	0.485	0.500	0.492
**TFIDF-NB**	0.970	0.916	0.485	0.500	0.492
**TFIDF-LogReg**	0.972	0.922	0.948	0.540	0.567
**TFIDF-SVM**	0.976	0.937	0.879	0.631	0.691
**Bi-LSTM**	0.975	0.942	0.820	0.680	0.728
**BERT-SVM**	0.970	0.916	0.485	0.500	0.492
**BERT-CNN**	0.970	0.916	0.485	0.500	0.492
**BERT-NN**	0.978	0.952	0.832	0.746	0.782

Table 4: Metrics for Religion

	Accuracy	Accuracy (hate only)	Precision	Recall	F1
**TFIDF-NN**	0.829	0.528	0.578	0.504	0.566
**TFIDF-NB**	0.833	0.528	0.917	0.505	0.464
**TFIDF-LogReg**	0.910	0.766	0.899	0.759	0.806
**TFIDF-SVM**	0.920	0.820	0.874	0.825	0.847
**Bi-LSTM**	0.915	0.821	0.857	0.830	0.843
**BERT-SVM**	0.933	0.880	0.871	0.897	0.884
**BERT-CNN**	0.907	0.756	0.893	0.754	0.801
**BERT-NN**	0.932	0.880	0.869	0.897	0.882

Table 5: Metrics for Race

	Accuracy	Accuracy (hate only)	Precision	Recall	F1
**TFIDF-NN**	0.959	0.889	0.480	0.499	0.490
**TFIDF-NB**	0.961	0.889	0.480	0.500	0.490
**TFIDF-LogReg**	0.963	0.900	0.805	0.550	0.579
**TFIDF-SVM**	0.963	0.901	0.785	0.565	0.600
**Bi-LSTM**	0.964	0.910	0.777	0.613	0.657
**BERT-SVM**	0.961	0.889	0.480	0.500	0.490
**BERT-CNN**	0.961	0.889	0.480	0.500	0.490
**BERT-NN**	0.969	0.930	0.808	0.729	0.762

Table 6: Metrics for Gender

	Accuracy	Accuracy (hate only)	Precision	Recall	F1
**TFIDF-NN**	0.927	0.797	0.536	0.501	0.486
**TFIDF-NB**	0.930	0.800	0.965	0.506	0.493
**TFIDF-LogReg**	0.957	0.896	0.898	0.745	0.800
**TFIDF-SVM**	0.965	0.927	0.892	0.825	0.855
**Bi-LSTM**	0.963	0.937	0.861	0.859	0.860
**BERT-SVM**	0.970	0.952	0.889	0.888	0.888
**BERT-CNN**	0.947	0.865	0.882	0.664	0.723
**BERT-NN**	0.972	0.957	0.887	0.905	0.896

Table 7: Metrics for GI/SO

	Accuracy	Accuracy (hate only)	Precision	Recall	F1
**TFIDF-NN**	0.978	0.942	0.490	0.499	0.494
**TFIDF-NB**	0.980	0.942	0.490	0.500	0.495
**TFIDF-LogReg**	0.981	0.948	0.900	0.544	0.576
**TFIDF-SVM**	0.982	0.953	0.849	0.598	0.650
**Bi-LSTM**	0.975	0.955	0.697	0.716	0.706
**BERT-SVM**	0.980	0.942	0.490	0.500	0.495
**BERT-CNN**	0.980	0.942	0.489	0.5	0.494
**BERT-NN**	0.983	0.959	0.823	0.671	0.723

Table 8: Metrics for Immigration

	Accuracy	Accuracy (hate only)	Precision	Recall	F1
**TFIDF-NN**	0.890	0.695	0.446	0.499	0.471
**TFIDF-NB**	0.893	0.697	0.946	0.501	0.474
**TFIDF-LogReg**	0.895	0.707	0.793	0.518	0.508
**TFIDF-SVM**	0.905	0.739	0.841	0.581	0.612
**Bi-LSTM**	0.899	0.764	0.733	0.673	0.697
**BERT-SVM**	0.914	0.758	0.875	0.619	0.665
**BERT-CNN**	0.893	0.696	0.446	0.500	0.472
**BERT-NN**	0.917	0.811	0.790	0.743	0.764

Table 9: Metrics for E/I/N

	Accuracy	Accuracy (hate only)	Precision	Recall	F1
**TFIDF-NN**	0.912	0.754	0.502	0.500	0.479
**TFIDF-NB**	0.914	0.756	0.457	0.500	0.477
**TFIDF-LogReg**	0.947	0.858	0.943	0.708	0.775
**TFIDF-SVM**	0.967	0.921	0.939	0.844	0.884
**Bi-LSTM**	0.970	0.942	0.911	0.895	0.903
**BERT-SVM**	0.975	0.951	0.933	0.907	0.920
**BERT-CNN**	0.914	0.756	0.457	0.500	0.477
**BERT-NN**	0.978	0.959	0.933	0.928	0.931

Table 10: Metrics for Antisemitism

Focusing on the BERT-NN model architecture, Figure 2 allows us to compare performance across types of hate speech. Accuracy is shown as the percentage of the 4969 test posts correctly classified by the machine. Accuracy ranged from 98.3% for immigration to 91.7% for E/I/N. Figure 3 shows the F1 score for each of these models. On this metric, performance was best for antisemitism and lowest for immigration hate speech. Finally, because the test data set is imbalanced (i.e., for any given hate speech type, most posts do not contain that type of hate speech), we analyze accuracy while focusing only on posts that were coded by human annotators as containing a given type of hate speech. For each type of hate speech, Figure **??** shows the percentage of posts containing that type of hate speech that were correctly coded by the machine. Accuracy along this metric remains relatively high for most hate speech types, although there are significantly more false-negatives with respect to E/I/N.

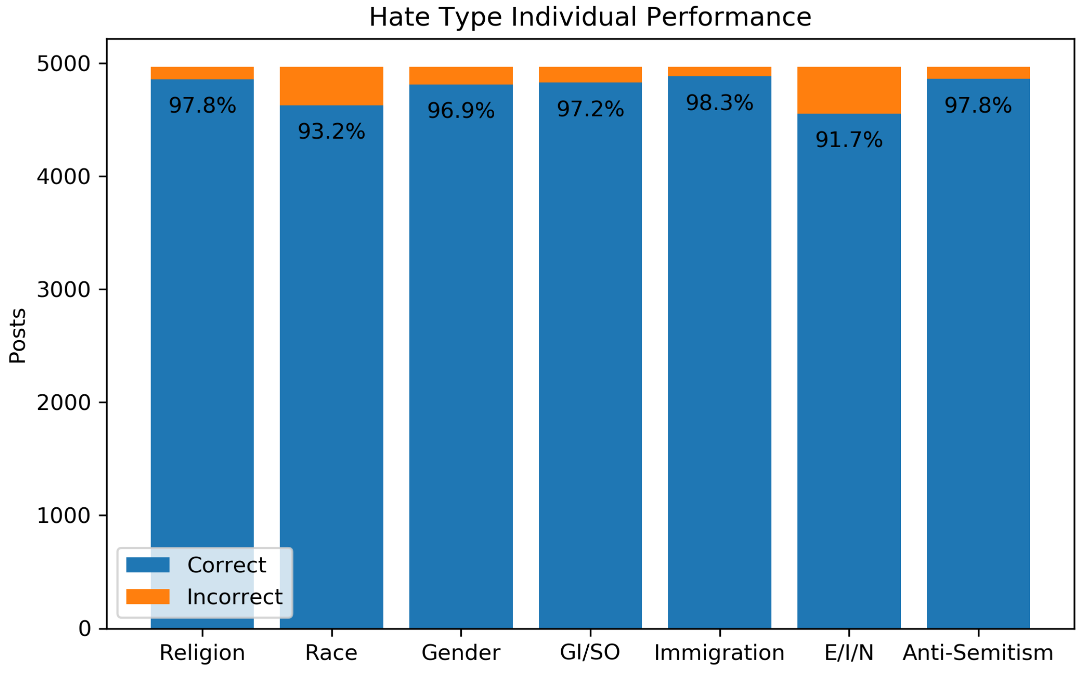

Figure 2: Machine Performance for Each Type of Hate Speech - Accuracy

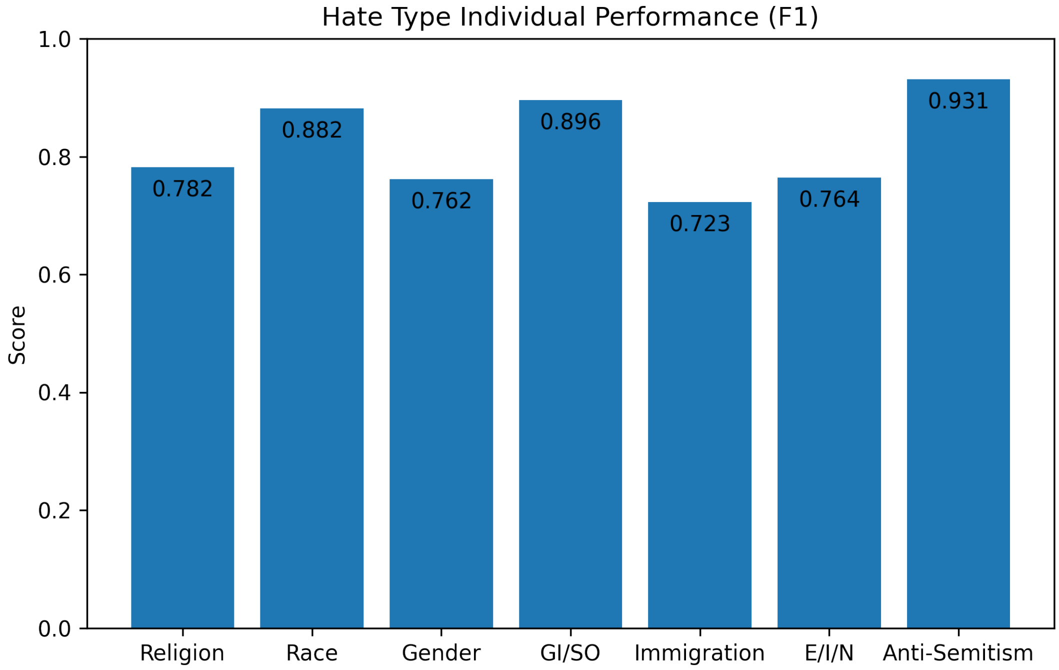

Figure 3: Machine Performance for Each Type of Hate Speech - F1

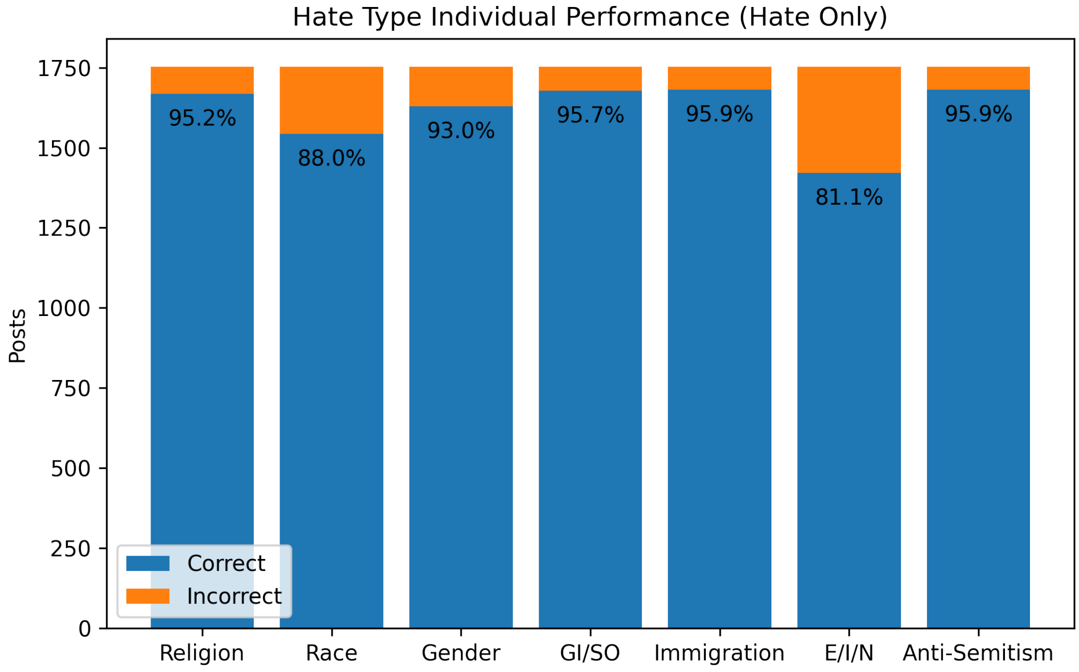

Figure 4: Machine Performance for Each Type of Hate Speech - Accuracy - True Positives Only

We conducted a validation analysis of the machine coding that is substantially similar to the inter-coder reliability analysis discussed above. Each of Coders A and B re-coded a different random sample of 1000 posts initially coded by the machine. Our analysis of these results is shown in Tables 11 and 12. With respect to the re-coding by Coder A, Cohen's Kappa ranges from 0.83 to 1, depending on the type of hate speech. With respect to the re-coding by Coder B, Cohen's Kappa ranges from 0.85 to 0.94. All of these values are above the standards used by the literature, indicating that the machine coding is highly reliable.

	**Agreement**	**Expected Agmnt.**	**Kappa**	**SE**	**Z**	**p>Z**
**Religion**	99.80%	97.43%	0.9221	0.0316	29.16	0.0000
**Race**	98.00%	78.57%	0.9067	0.0316	28.72	0.0000
**Gender**	99.50%	96.95%	0.8362	0.0316	26.46	0.0000
**GI/SO**	99.30%	90.23%	0.9283	0.0316	29.36	0.0000
**Immigration**	100.00%	99.40%	1.0000	0.0316	31.62	0.0000
**E/I/N**	98.90%	88.63%	0.9033	0.0316	28.62	0.0000
**Anti-Semitism**	98.60%	85.11%	0.9060	0.0316	28.66	0.0000

Table 11: Machine Coding Reliability - Coder A

	Agreement	Expected Agmnt.	Kappa	SE	Z	p>Z
**Religion**	99.70%	97.13%	0.8950	0.0315	28.42	0.0000
**Race**	98.40%	80.72%	0.9170	0.0315	29.07	0.0000
**Gender**	99.60%	97.04%	0.8646	0.0316	27.41	0.0000
**GI/SO**	99.50%	91.68%	0.9399	0.0316	29.74	0.0000
**Immigration**	99.90%	99.30%	0.8567	0.0313	27.37	0.0000
**E/I/N**	98.70%	90.95%	0.8564	0.0315	27.16	0.0000
**Anti-Semitism**	98.70%	87.93%	0.8923	0.0316	28.24	0.0000

Table 12: Machine Coding Reliability - Coder B

4 Automated Topic Analysis

Our manual analysis suggests the 4 largest increases in online hate posts are associated with specific offline events. Two of these offline events were well-known and highly publicized: the death of George Floyd/BLM protests, and the U.S. Election. The others – the assassination of Iranian General Qasem Soleimani and the crisis at the border of Turkey and Greece – were much less generally well-known. Thus, we use automated topic analysis to confirm that the following spikes in online hate speech were related to these events: (1) the spikes in religion and anti-semitism hate speech in early January 2020; and (2) the spike in immigration hate speech in late February / early March 2020.

We use an unsupervised machine learning technique called Latent Dirichlet Allocation (LDA) to analyze the conversation topics around spikes in hate types. The LDA method models documents (in our case, social media posts) as distributions of topics and topics as distributions of words. During its training process, these distributions are adjusted to fit the data. The LDA method creates groups of words – "topics" – based on how they tend to be used together in context. It requires one input parameter: the number of topics to find (n_topics), but otherwise it is entirely unsupervised.

To evaluate the "goodness of fit" for a particular LDA model we use C_V coherence. The coherence score provides a quantitative method for measuring the alignment of the words within an identified topic. It is generated from a separate algorithm which is run over a trained LDA model. The overall coherence score of a single model is the arithmetic mean of its per-topic coherences. There are many different coherence metrics to evaluate per-topic coherence. We use C_V, which is common in the field.

For each of the two relevant spikes in online hate speech, we identified the posts classified by our algorithm as containing the relevant type of hate during the relevant dates. For each set of posts, we generate 10 LDA models for each n_topics $\in [3, 15]$. We average the coherence scores for each of these models and use the highest scoring model for the n_topics with the highest average coherence score.

The keyword distributions for the highest-coherence topic surrounding each hate speech spike are shown in Figures 5 through 7. Figure 5 shows the top keywords in the topic with the largest coherence score among the posts that contained anti-semitism following the Soleimani assassination. While words such as Israel and Zion are common among anti-semitic posts in general, several other top keywords here are much less common at other times, including Iran, war, support, and master. The use of these words reflects the narrative in these posts, i.e., the false notion that Israel masterminded the assassination in order to provoke a war between Iran and the West. As noted in the main text, the religion hate speech following this event consisted mostly of Islamophobia. As shown in Figure 6, the top keywords in the topic with the largest coherence score among the relevant posts include several that are relevant to the assassination, including: shia (Soleimani's religion), attack, kill, and Iran, which are not regularly among the top words in religion hate speech.

As shown in Figure 7, religion hate posts at the time of the Greece-Turkey border crisis include several top keywords that are directly related to that event, including greek, greec, turkish, turkey, and europ. By contrast, discussion of more typical immigration hate speech topics such as the US-Mexico border, is largely not reflected in these posts.

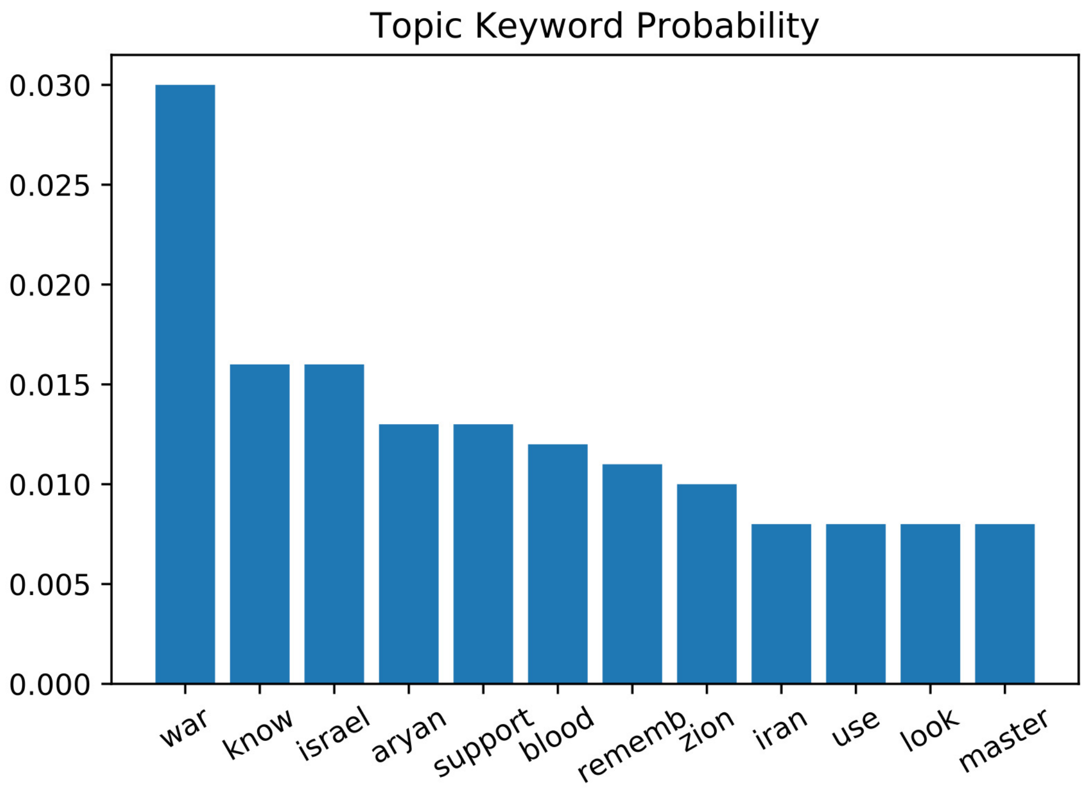

Figure 5: Anti-semitism and the Soleimani assassination. Topic coherence: 0.878

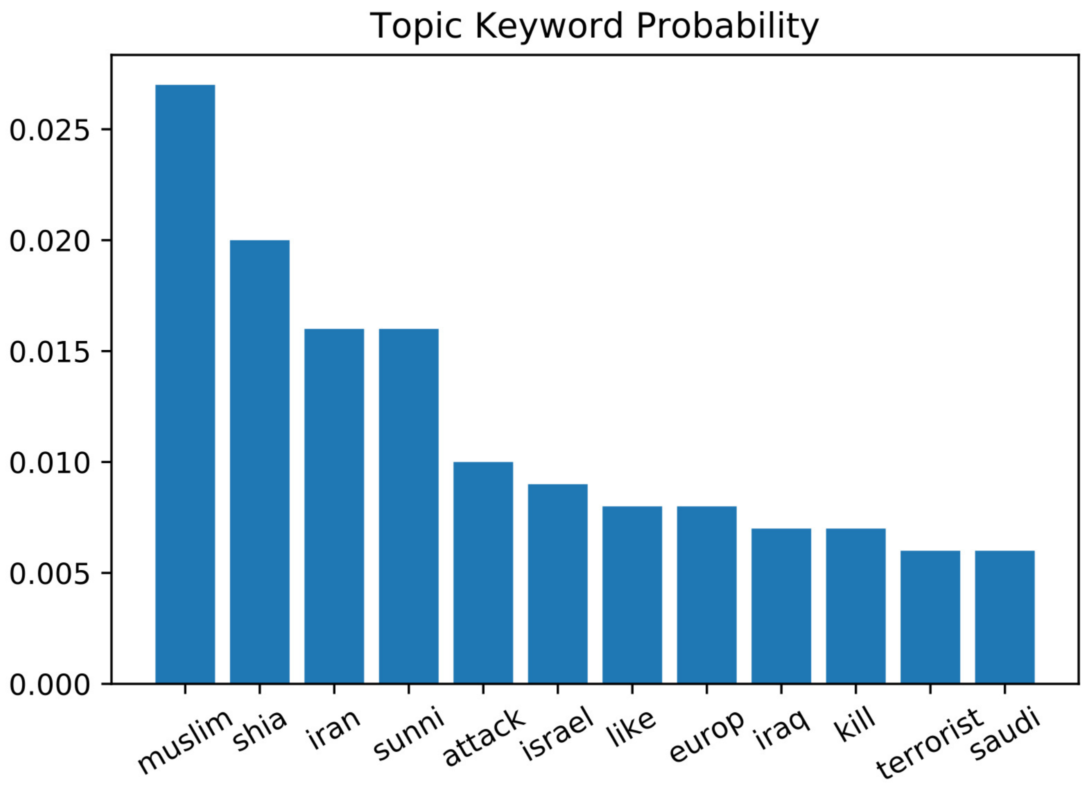

Figure 6: Religious hate and the Soleimani assassination. Topic coherence: 0.676

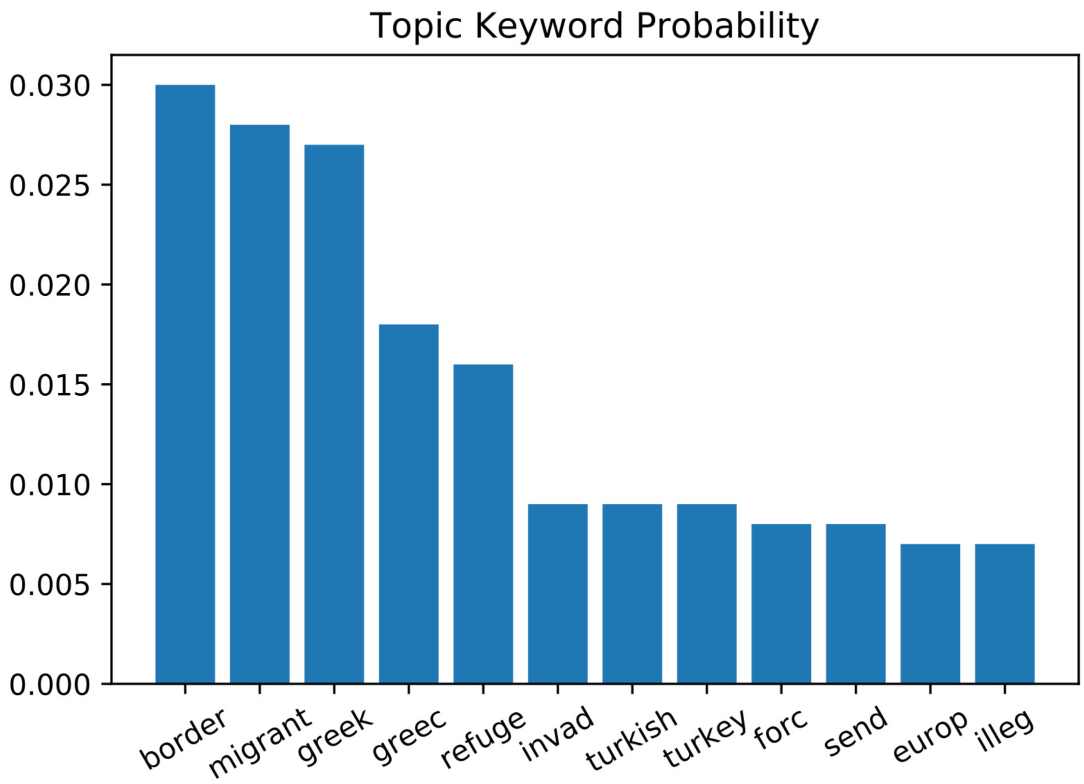

Figure 7: Immigration hate and the Greece-Turkey border crisis. Topic coherence: 0.771

5 Additional Results

This section provides additional information regarding our data on a platform-by-platform basis. Figures 8 through 13 show the rolling averages of the daily posts, hate posts, percentages of posts that contain hate, and average number of hate speech types in hate posts, each by platform for the duration of the study period. Gab experienced a series of server crashes between late March and late May of 2020, resulting in an interruption to our data capture. As a result, our data set includes very few posts from Gab during that period. A similar interruption occurred on Gab in the second half of December, 2020. Figures 14 through 19 show, for each platform, the temporal trends in each type of hate speech over the study period.

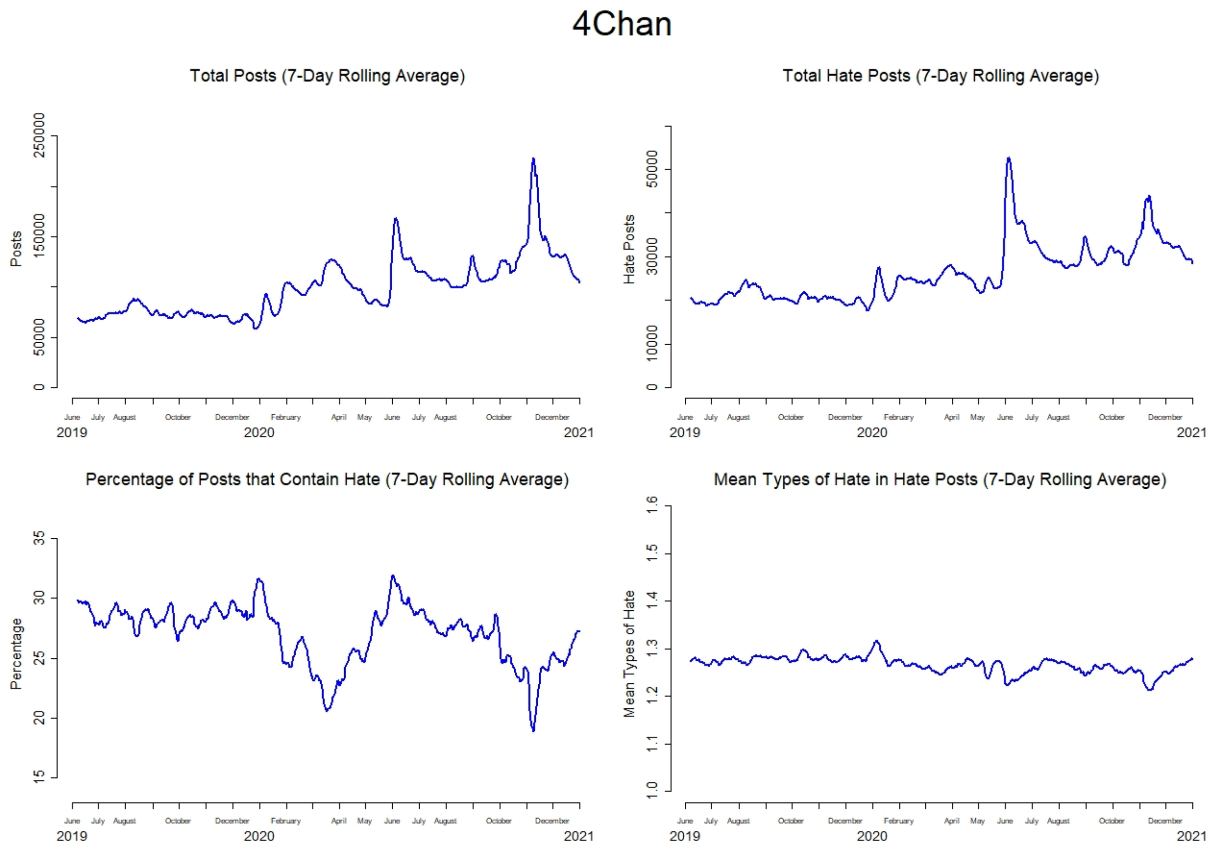

Figure 8: Summary of 4Chan Posts over Time

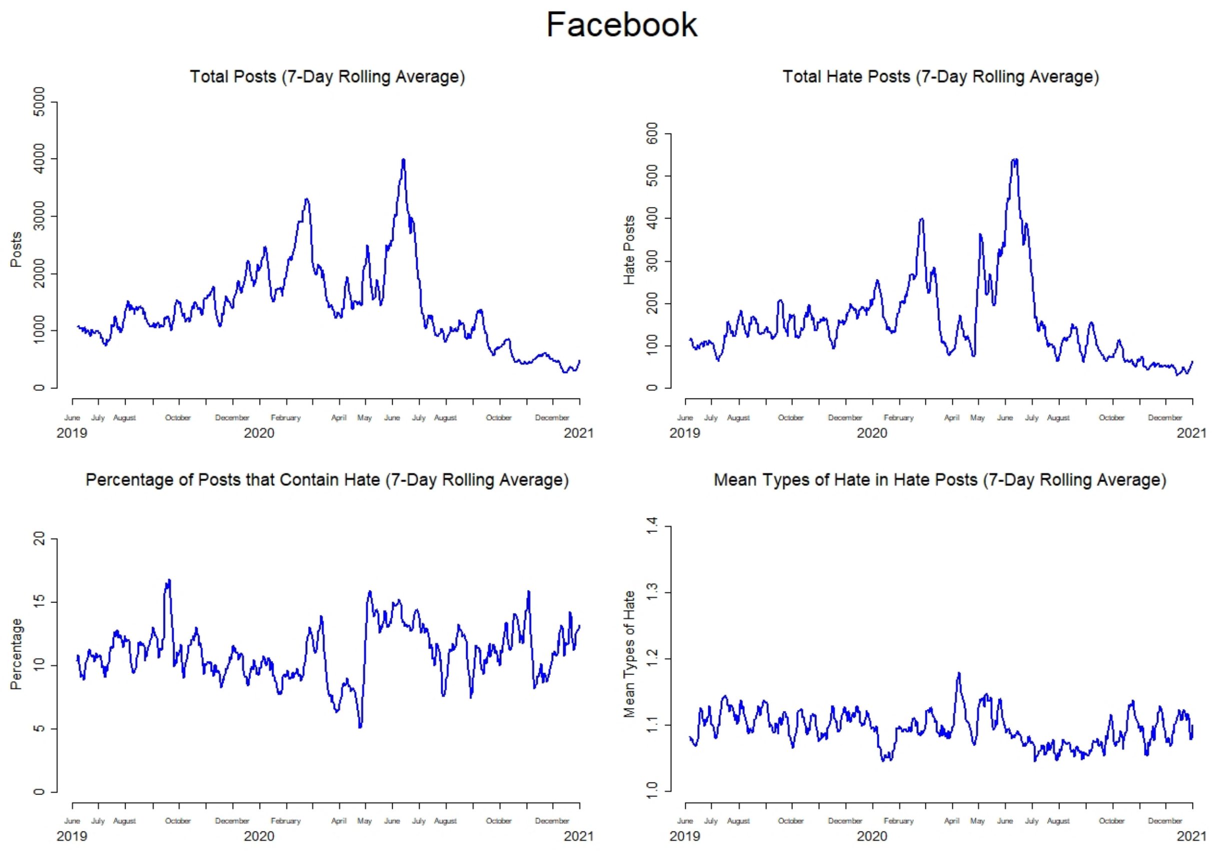

Figure 9: Summary of Facebook Posts over Time

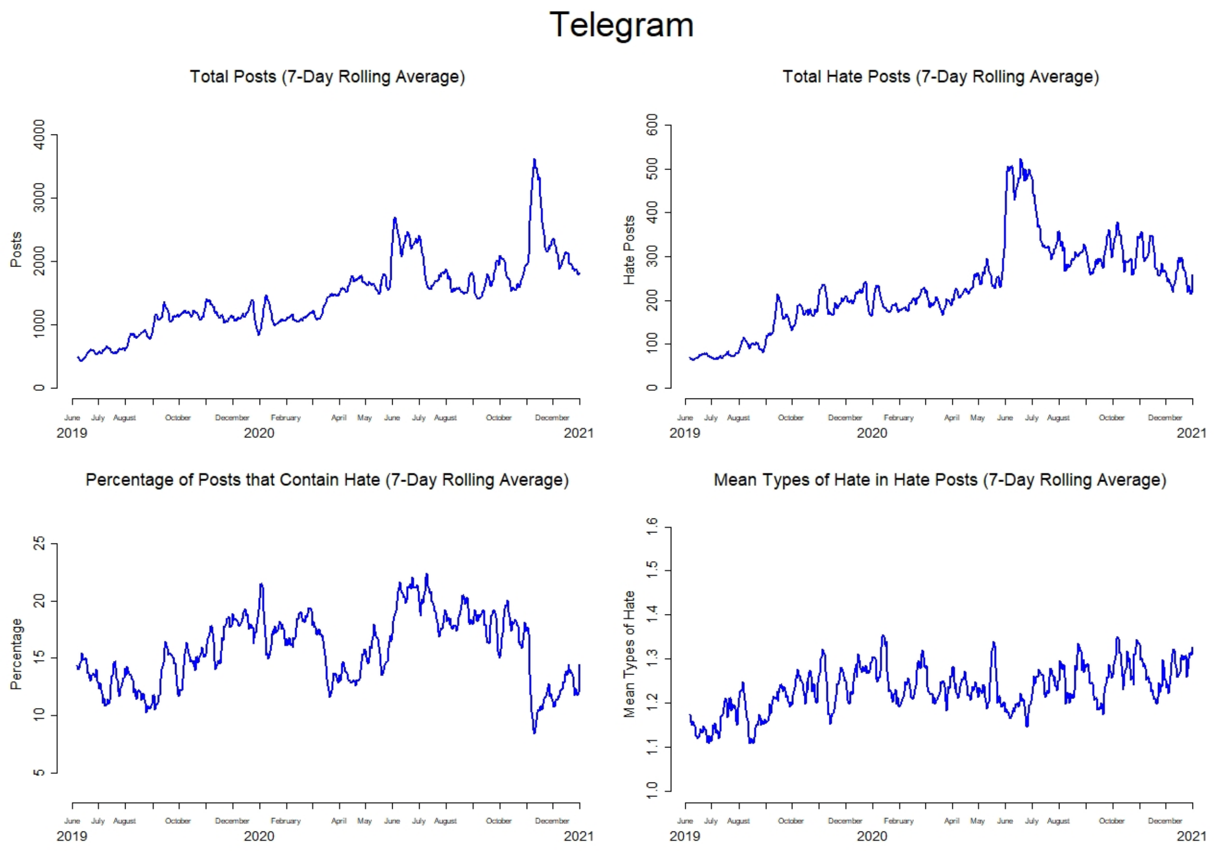

Figure 10: Summary of Telegram Posts over Time

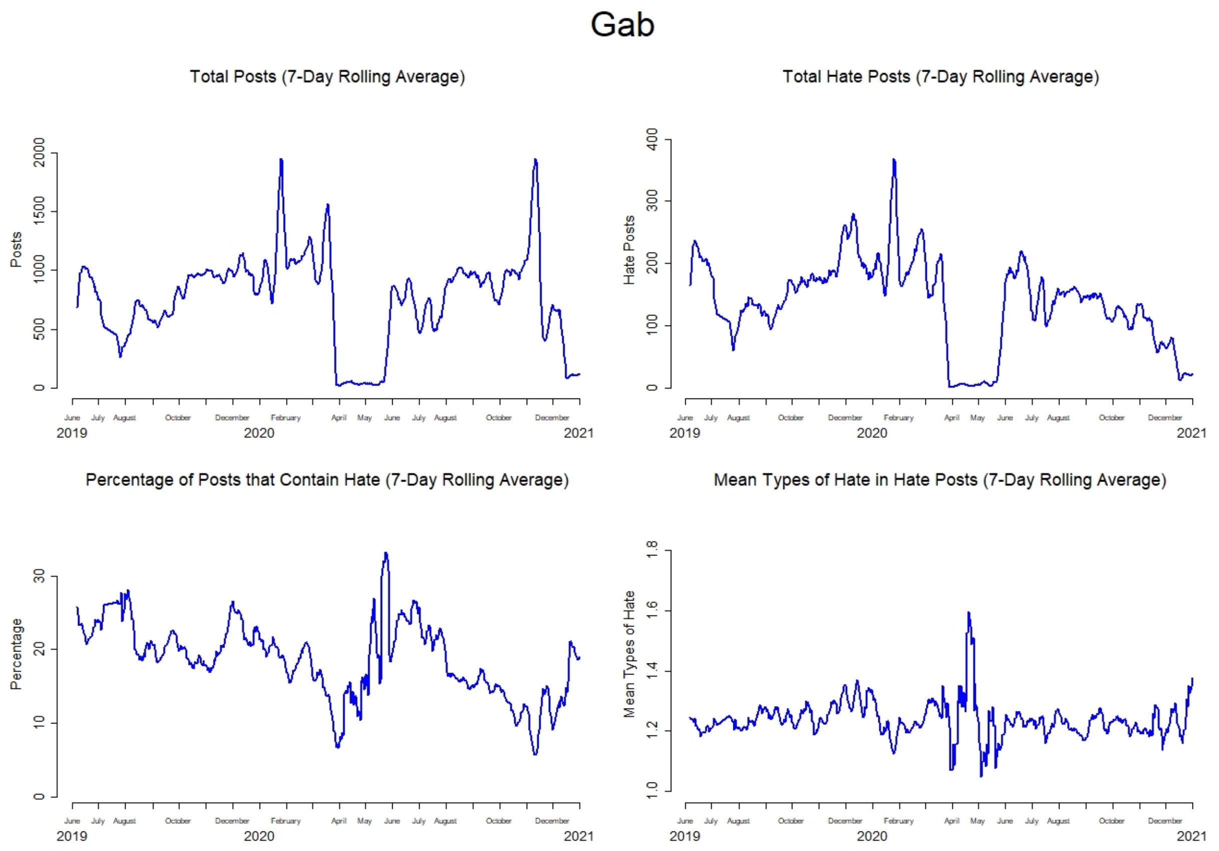

Figure 11: Summary of Gab Posts over Time

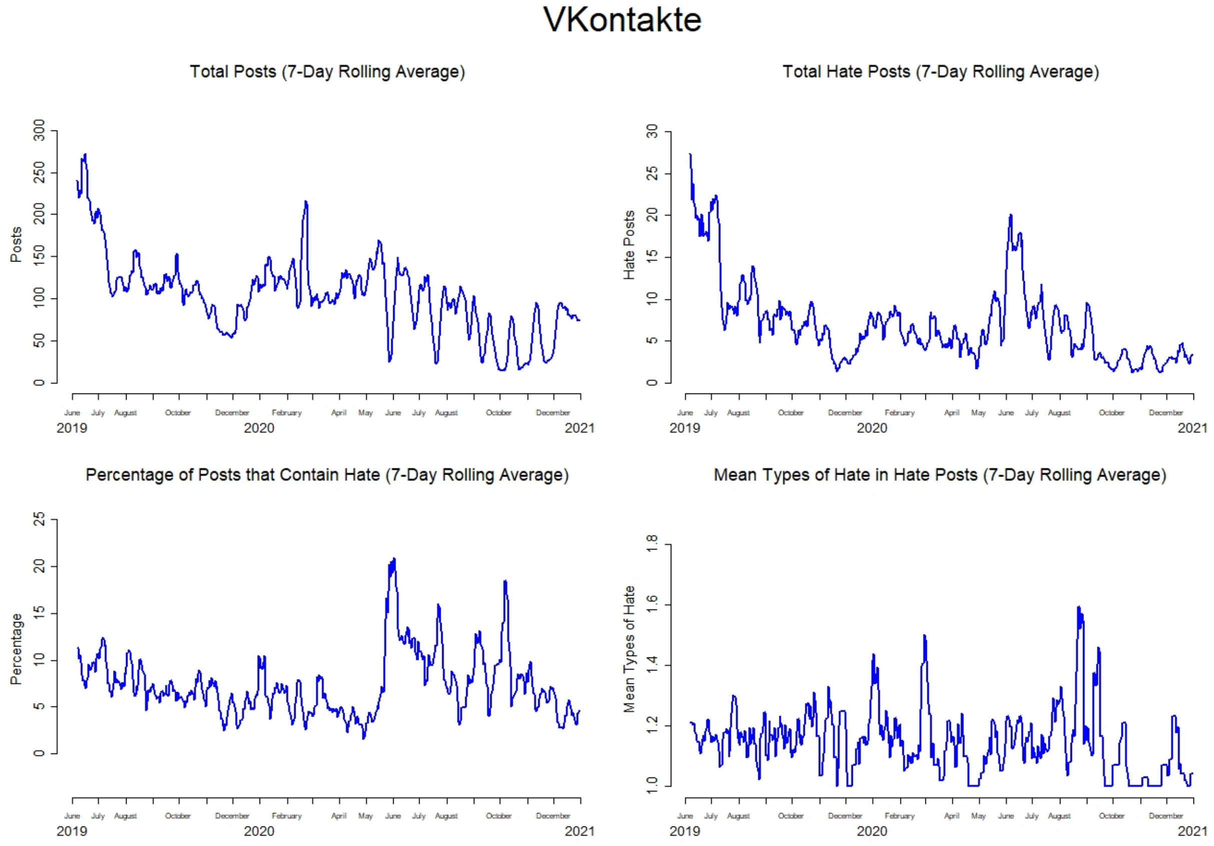

Figure 12: Summary of VKontakte Posts over Time

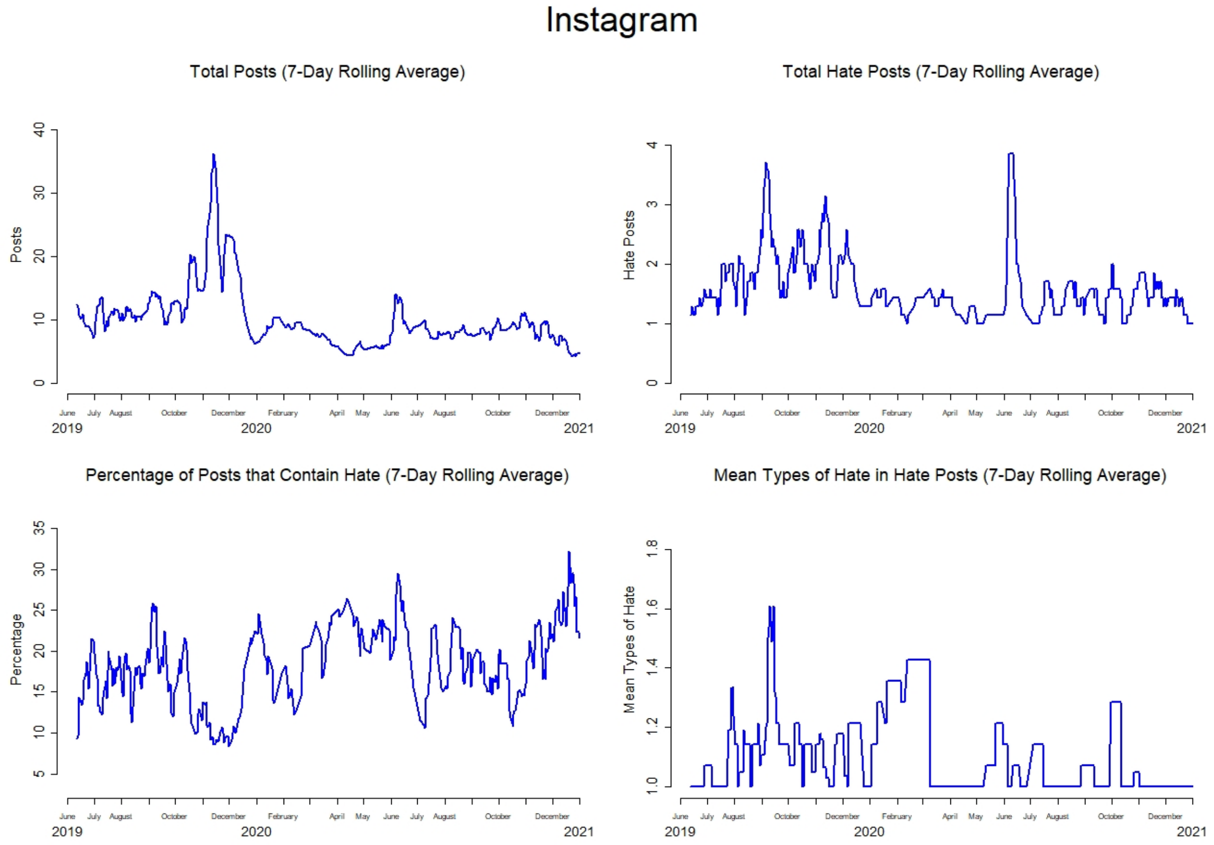

Figure 13: Summary of Instagram Posts over Time

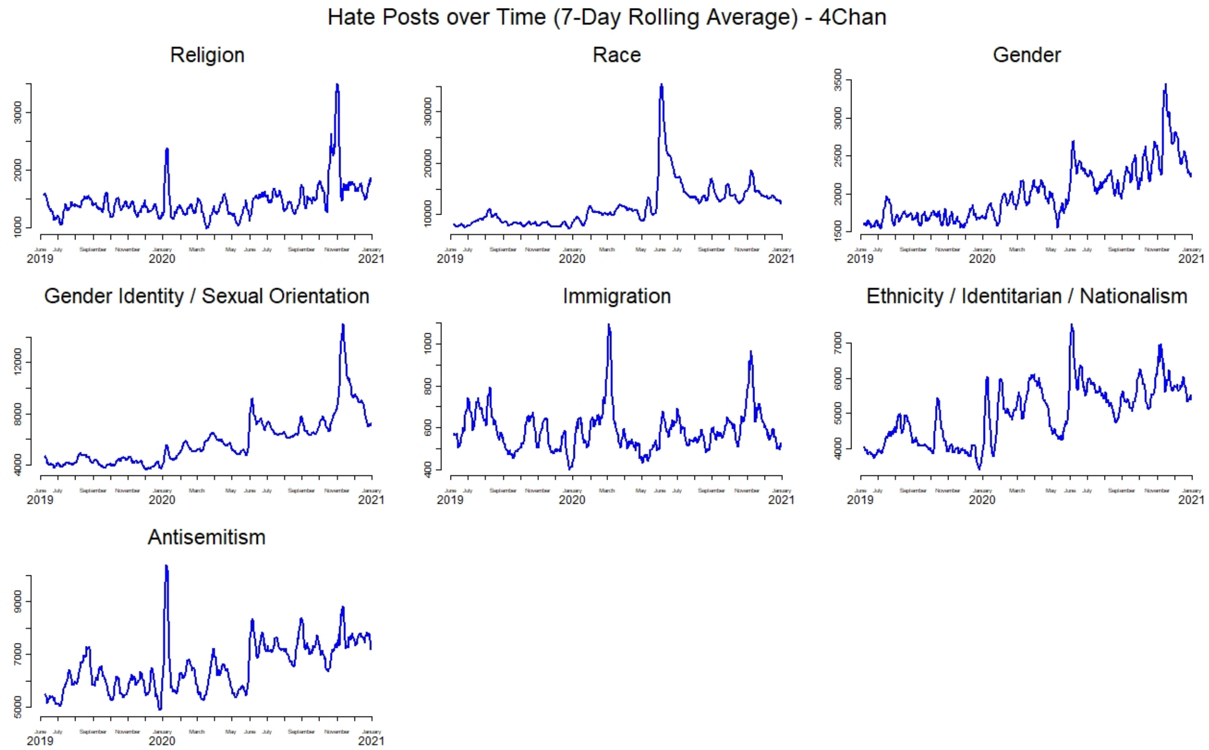

Figure 14: Hate Speech Types on 4Chan over Time

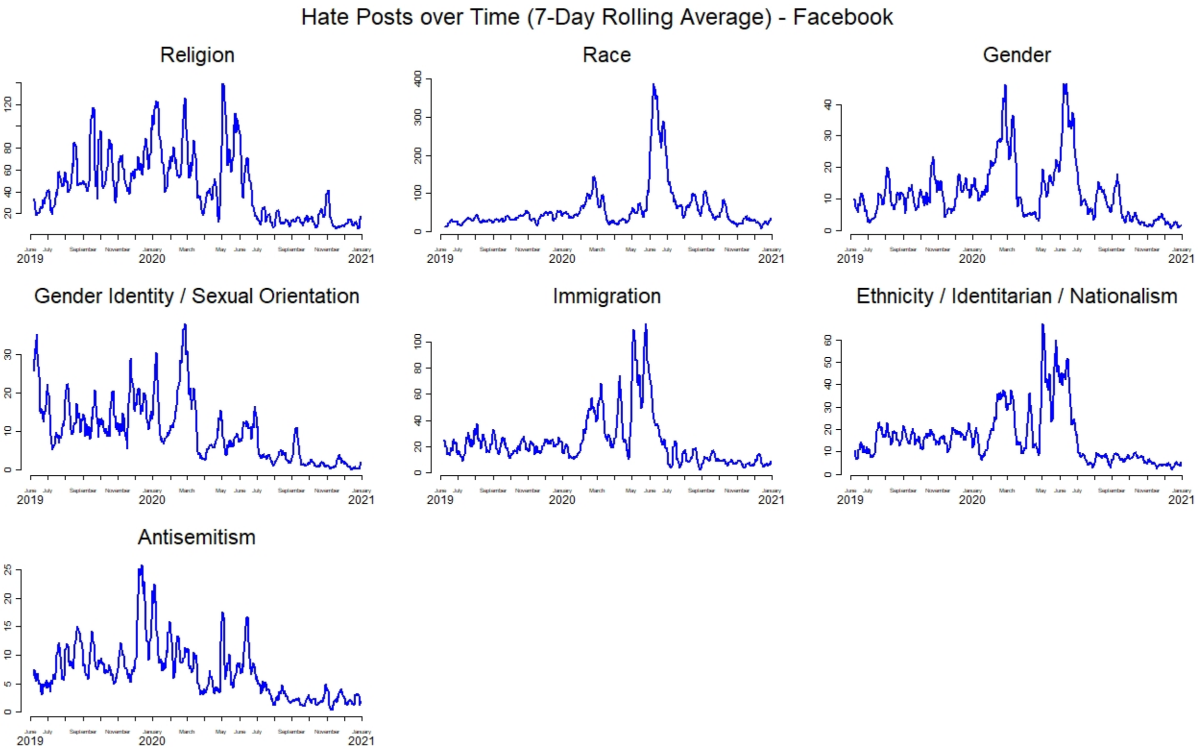

Figure 15: Hate Speech Types on Facebook over Time

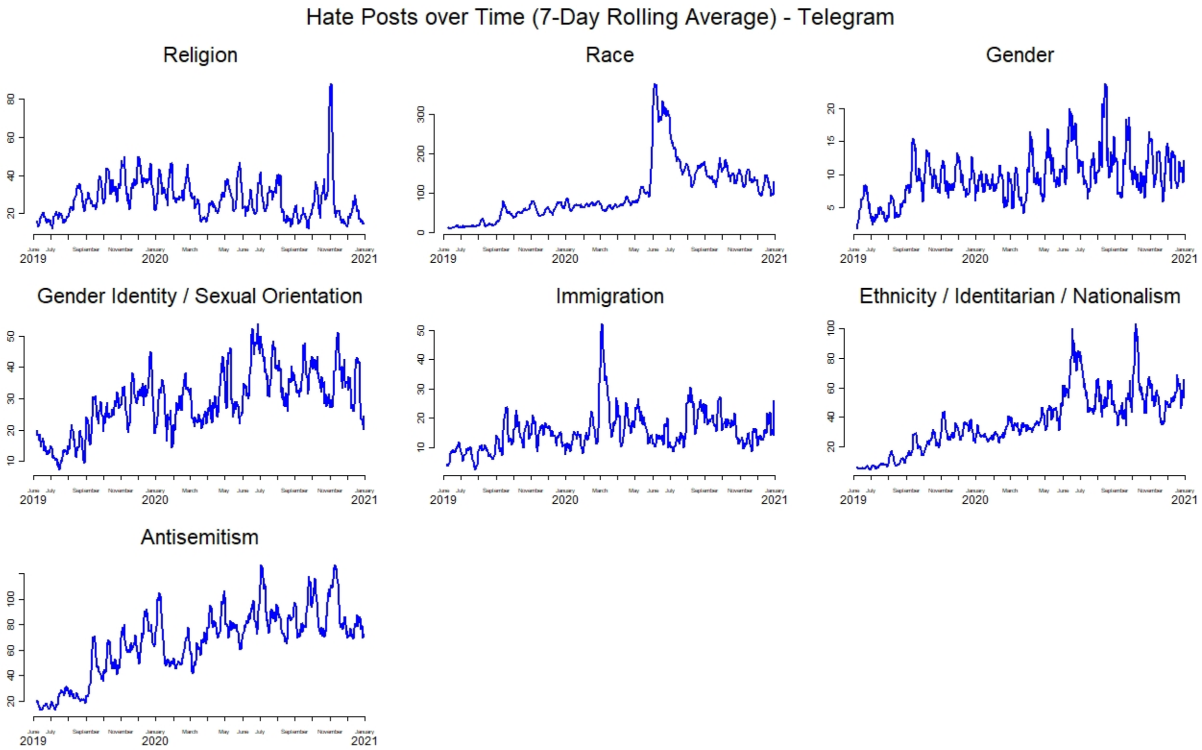

Figure 16: Hate Speech Types on Telegram over Time

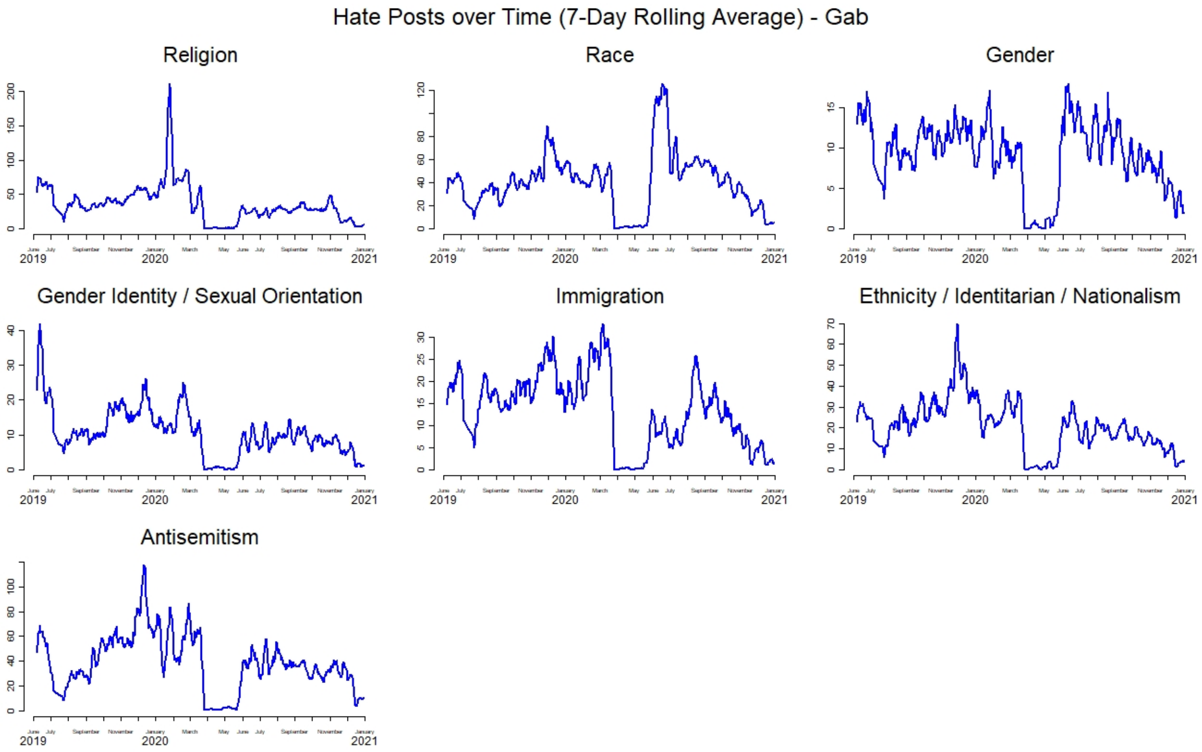

Figure 17: Hate Speech Types on Gab over Time

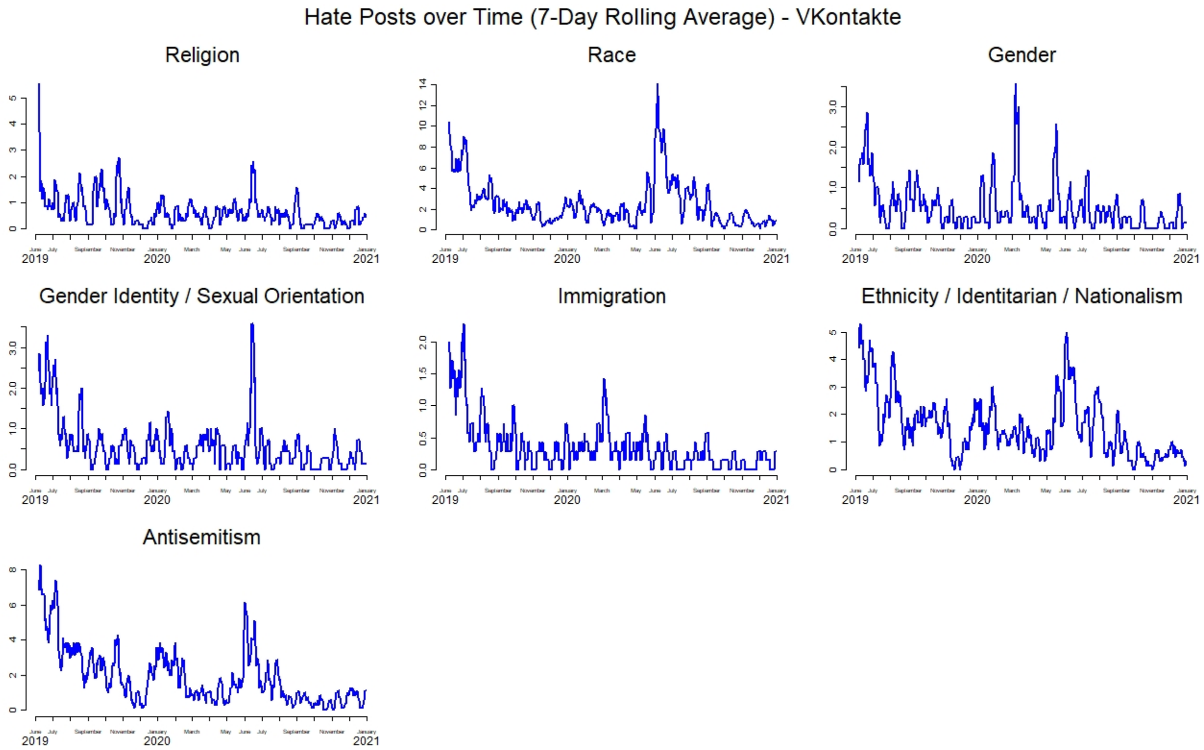

Figure 18: Hate Speech Types on VKontakte over Time

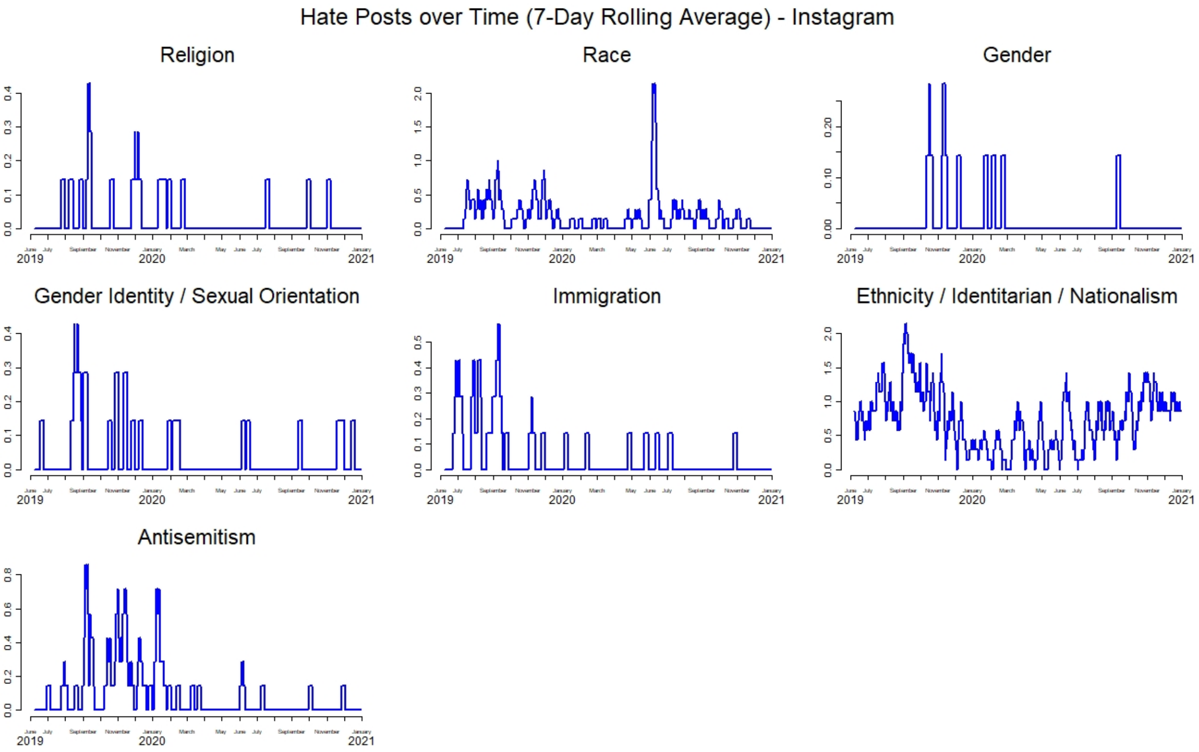

Figure 19: Hate Speech Types on Instagram over Time

6 References

[1] Mann, M. (2004). Fascists. Cambridge University Press.

[2] Hine, G. E., Onaolapo, J., De Cristofaro, E., Kourtellis, N., Leontiadis, I., Samaras, R., and Blackburn, J. (2017, May). Kek, cucks, and god emperor trump: A measurement study of 4chan's politically incorrect forum and its effects on the web. In Eleventh International AAAI Conference on Web and Social Media.

[3] Lagorio-Chafkin, C. (2018). We are the nerds: The birth and tumultuous life of Reddit, the internet's culture laboratory. Hachette UK.

[4] Sechidis, K., Tsoumakas, G., and Vlahavas, I. (2011, September). On the stratification of multi-label data. In Joint European Conference on Machine Learning and Knowledge Discovery in Databases (pp. 145-158). Springer, Berlin, Heidelberg.

[5] Lee, J. Y., and Dernoncourt, F. (2016). Sequential Short-Text Classification with Recurrent and Convolutional Neural Networks. In Proceedings of NAACL-HLT (pp. 515-520).

[6] Wolf, T., Chaumond, J., Debut, L., Sanh, V., Delangue, C., Moi, A., ... and Rush, A. M. (2020, October). Transformers: State-of-the-art natural language processing. In Proceedings of the 2020 Conference on Empirical Methods in Natural Language Processing: System Demonstrations (pp. 38-45).
