# Supplementary material for: Offline events and online hate
Source: PLoS One. 2023 Jan 25;18(1):e0278511. doi: 10.1371/journal.pone.0278511 (PMC9876356; doi:10.1371/journal.pone.0278511)
Supplement: Attachment — Submitted filename: Plos One Response Memo.docx [file pone.0278511.s002.docx]

**Response Memo for Revisions to “Offline Events and Online Hate” (PONE-D-22-01701)**

We would like to thank the editors and reviewers for their extremely helpful comments. We believe these suggestions have made for a stronger overall paper. Below, we outline the changes we made with reference to specific comments. As in the original submission, a set of supplementary appendices accompanies the main paper.

**Response to the Editors’ Comments:**

1. “Please ensure that your manuscript meets PLOS ONE's style requirements, including those for file naming.”

We have revised the submission to comply with PLOS ONE’s requirements.

2. “Please provide additional details regarding participant consent. In the Methods section, please ensure that you have specified (1) whether consent was informed and (2) what type you obtained (for instance, written or verbal). If the need for consent was waived by the ethics committee, please include this information.

*In line with our policy regarding third-party data use (<https://journals.plos.org/plosone/s/submission-guidelines#loc-personal-data-from-third-party-sources>), please provide a copy of the protocol that your IRB reviewed and approved, and which specifies what types of data you collected and how data were anonymized. Please submit this as an ‘Other’ file, as this will be only for our records and will not be published.

- please also ping me and the Data team at PRTC to discuss the data sharing/availability options for this ms.”

Thank you for raising this important concern. As noted in our email to the editors of May 17, 2022, our project was reviewed by GW's IRB and approved as "exempt".  This was because (a) we do not collect any personally identifying information; (b) all information we collected was posted on the public Internet; and (c) we do not interact with the individuals who posted that information in any way.  As instructed by the editorial assistant via email on May 23, 2022, we have included this information in this response. A copy of our IRB approval form has been submitted with our revised manuscript.

Finally, as instructed by the editorial assistant via email on May 23, 2022, we have disregarded the request to contact the Data team.

3. “We note that you have stated that you will provide repository information for your data at acceptance. Should your manuscript be accepted for publication, we will hold it until you provide the relevant accession numbers or DOIs necessary to access your data. If you wish to make changes to your Data Availability statement, please describe these changes in your cover letter and we will update your Data Availability statement to reflect the information you provide.”

If our paper is accepted for publication in PLOS ONE, we will make the relevant data available in whatever repository(ies) the editors prefer.

**Response to R1’s Comments**

1. “First, there are several aspects of the authors’ sampling approach that they should do more to explain. Why do the authors look at only one community on 4chan? This seems particularly odd given that the vast majority of the authors’ training data comes from 4chan. How might this bias the authors’ ability to detect hate speech across platforms? The majority of their training data also focuses on racist hate speech in particular, but is this an artifact of using almost entirely data from a single 4chan community? This likely limits the authors ability to detect other hate speech types in their data, particularly across platforms. More generally, what is the advantage in focusing on hate groups rather than representative samples of posts across each platform (see Siegel et al. 2021). The authors need to do more to justify this approach.”

This is an important set of issues, and we thank R1 for highlighting it.

Focus on hate communities. We focus on online hate communities largely because of the sparsity of hate speech on most online communities. To perform our analyses we needed a data set that contained many examples of each of the 7 types of hate speech we studied. It was also important for these examples to reflect a wide range of word choices for each of these 7 types of hate speech. In other words, we needed a data set that contained a large and diverse set of examples of hate speech.

Especially on moderated platforms, hate speech has (thankfully!) become less frequent on all but a few communities. Even on fringe platforms, users who espouse hateful views tend to congregate on relatively few communities, perhaps because they seek to communicate with others who share their views and likely also because of the potential for moderation elsewhere (e.g., 4Chan, as explained below). It would therefore not have been practical for us to collect a sufficient set of observations that contained hate speech at the scale of this project without first focusing on the communities in which it is consistently used. We now discuss this issue on page 1 of the SI.

For example, to give a sense of the sparsity of online hate speech outside of these hate communities, our team analyzed a data set of ~2000 Facebook communities that are not associated with hate speech from 2017 to 2019. These are data we have from an earlier project. Our algorithm found that approximately 0.06% of the posts in these data contained hate speech.

Unfortunately, there is no practical way of collecting random sample data on the platforms we studied. This is possible on Twitter, which was the platform studied in the excellent article by Siegel et al. 2021 referenced by R1. As noted above, even if we could access such a random sample, the frequency of hate speech would likely be too sparse for our purposes. In fact, the authors of that paper supplemented their Twitter data with a corpus of data from communities on Reddit that were known for using large volumes of hate speech (and that have since been removed from Reddit). They did so for reasons that seem similar to the motivations behind our data collection effort: to have a corpus in which hate speech is known to be used frequently.

4Chan. Perhaps the most well-known of the communities in our data is the Politically Incorrect forum on 4Chan. This forum is perhaps the most important and influential hate community on the Internet, and most likely its most active. Politically Incorrect, or “/pol/” is known as both the central gathering place for online hate and extremism and as a forum in which users believe they can speak freely without fear of moderation. The forum was created in order to centralize and contain the hate content on 4Chan, so that it can be kept off of other forums on the site. 4Chan users often make explicit reference to the notion that they could not say what they say on /pol/ elsewhere on 4Chan without fear of content moderation. In terms of 4Chan, therefore, this is why we included only 1 community. We now discuss this issue on page 2 of the SI.

R1 also asks whether the prevalence of 4Chan data might bias our ability to detect hate speech elsewhere. Indeed, users on different platforms tend to use language differently. This manifests also in their use of hate speech. Perhaps the most striking example we found of this is the frequent use of the “n-word” on 4Chan, where that word is not moderated. By contrast, this word is immediately moderated on Facebook and therefore appears in 0 observations in our data from Facebook. Yet Facebook users can and do express racist hate speech using other terms. As we note in lines 324-327, in percentage terms racist hate speech increased more on Facebook after the death of George Floyd than on other platforms.

This is why we made sure to collect large numbers of posts across the platforms. The only platform in which this provided difficult was Instagram, from which we collected about 5000 posts. We spent significant effort searching for other hate communities on Instagram and were not able to find any additional ones, likely because the platform has been effective in removing hate speech. We now discuss this issue on page 1 of the SI.

Finally, R1 asks about the prevalence of racist hate speech from 4Chan in our data set. There are two potential issues here. First is whether the fact that we have more posts that contain racist hate speech than other types of hate speech is driven by the prevalence of 4Chan in our data set. In fact, on each platform, racist hate speech was the most common type of hate speech in our data set.

Second is whether having more posts with racist hate speech biases our ability to detect other types of hate speech. Given that each hate speech type is classified by a separate algorithm trained only on the manual classification of that hate speech type, there is no reason for us to think the volume of any other hate speech type would create bias. In other words, for example, the algorithm that classifies anti-semitism only learns which posts were classified as containing that type of hate speech and which were not – it does not learn anything about racist hate speech. The question is whether we have a sufficient volume of posts containing each type of hate speech in our training data to sufficiently train the algorithm to classify it. (Ensuring this goes back to the motivation for our sampling approach, discussed above). We note that the type of hate speech least common in our training data is immigration, with 840 posts. The validation exercise discussed on pages 16-17 of the SI goes toward understanding whether we have sufficient posts that contain hate speech of each type to train the machine. With respect to immigration, the Cohen’s Kappa for Coder A is 1, and for Coder B it is 0.85 (Tables 11 and 12 in the SI), both indicating a highly reliable machine coding. Overall, given the results of that validation exercise, we do not see an indication that the machine-coding of racist hate speech is more reliable than for other types of hate speech.

2. “Second, given how rare many types of hate speech are in the authors’ data, it seems the classifier performance metrics (often in the high 90s) are likely inflated. I would like to see balanced accuracy metrics to better understand whether high classifier performance is simply an artifact of having so many 0s in the data. The authors should also conduct systematic validation exercises, taking random samples of their data and using human coding to determine how accurate their classifier performance is in practice.”

We thank R1 for these suggestions. In the revised submission, we have included the results of a validation exercise, in which two human annotators each re-coded samples of 1000 posts coded by the machine. We found the machine results to be highly reliable. This is referenced in lines 144-149 of the text and described in greater detail on pages 16-17 of the SI.

Also in the revised SI, we provide not only the accuracy results, but also the accuracy for posts that contain hate speech, the precision, recall, and F1. These results are provided for each of the 8 model architectures and for each of the 7 algorithms using those model architectures. These results can be found on pages 10-12 (Tables 4-10) of the SI.

3. “Third, the authors focus on measuring changes in volume over time makes it very difficult to interpret their results. In the aftermath of an event that makes a particular group identity more salient (eg George Floyd protests), we should expect to see large increases in discussions of that group (eg African Americans) online. This would lead to an increase in hate speech volume simply because the group is discussed more often and a certain percentage of posts about that group will always include hate speech online. Indeed, when the authors do look at their results in percentage terms, they yield very different findings. The authors should do more to describe changes in absolute vs relative terms in their data and interpret any results referencing absolute changes with caution.”

These are very helpful comments. We agree that, particularly in the types of communities we studied, when a group identity becomes more salient, this would lead to increased discussion of that group, and thus, in some instances, increases in hate speech in absolute terms.

Nonetheless, we think changes in absolute terms are substantively important for several reason. From a content moderation perspective, it is important to knew that there was, for example, an increase in absolute terms in racist hate speech on Facebook during the George Floyd protests. This suggests that content moderators on that platform were unable to contain hate speech on the platform during that event as well as they could prior to it. Similarly, it’s useful to know when there are not such absolute changes in hate speech on a given platform following salient offline events, because that might point to users migrating to another platform and/or to effective content moderation.

Second, and perhaps more importantly, while the absolute increase in racist hate speech during the George Floyd protests is fairly intuitive, not all the spikes in hate speech we found were as intuitive. For example, the George Floyd protests also saw a 75% increase in hate posts in our data attacking LGBTQ individuals, even though that group identity is much less clearly linked to the offline event (see pages 16, 22-23). We think it’s useful, for example, to know that an offline event that is clearly linked to one identity can trigger absolute increases in hate speech not just with respect to that identity but to other identities. Did the event make both identities salient, increasing hate in absolute terms because both groups were discussed more? Or did the event raise discussion of race, and thus racial hate speech, and thus LGBTQ hate, without increasing other discussion of the latter? Assessing these competing causal pathways is beyond the scope of this paper, but nonetheless this result (and similar increases with respect to other types of hate speech at that time) – even in absolute terms – raises many interesting questions for future work about the relationships between offline events and different types of online hate speech (see pages 22-23). Nonetheless, when we do discuss temporal trends in posts and hate posts in absolute terms, we have revised the discussion on page 11 to reflect R1’s concerns.

We are actually a bit unclear about how R1 is using “relative terms” in the sense that we are not certain what changes would be relative *to* in R1’s usage. Specifically, we’re not certain if R1 means (a) that a relative change in hate posts means a change in the percentage of total posts that are hate posts; or (b) that a relative change in hate posts means a change in hate posts in percentage terms relative to hate posts prior to an event. That said, we describe below how our manuscript handles both (a) and (b). We apologize for our lack of understanding on this and welcome any clarification.

Figure 2 allows us to consider changes in hate posts in relative terms using meaning (a) above. Panels A and B use absolute terms – i.e., the absolute number of posts (in Panel A) and the absolute number of hate posts (in Panel B). Both panels show, for example, large spikes in total posts and hate posts around the George Floyd protests and 2020 elections. In absolute terms, this is important to know, including for the reason described above. As we note in the revised paper on page 11, the increases in hate posts could in part be due to increased salience. Panel C gets at the relative terms. We can see there that during the George Floyd protests the percentage of posts that contained hate increased, whereas around the election this percentage decreased. This is quite interesting, as we note on page 12, because it indicates that these events had two quite different impacts on hate posts in relative terms (to other posts) despite both increasing them in absolute terms.

Figures 4 and 5 allow us to consider changes in hate posts in relative terms using meaning (b) above. Here we focus on the percentage change in hate posts following an event relative to the number of hate posts prior to an event. As discussed above, we think these changes in hate posts following offline events are substantively interesting even if they derive in part from identity salience because (a) some relative changes are less clearly linked to events that trigger salience of a given identity; (b) relative changes vary in magnitude; and (c) relative changes vary across platforms. All of these raise important questions for future academic work and for content moderators.

**Response to R2’s Comments**

1. “As far as it concerns the paper’s structure, the ‘Introduction’ title should be written (maybe it’s an oversight), and the Conclusion section should be added; also, it would be great to separate the data collection and the methods, making it clear that the methods are about the multi-label classification of posts.”

Thank you for these suggestions, which we have incorporated in the revised manuscript.

2. “Move the discourse of lines [67-69] right before “Second, by classifying …” at line 65, because you’re talking about the six platforms again.”

Thank you for this suggestion, which we have incorporated in the revised manuscript in lines 85-89.

3. “You could start the ‘Classification Method’ section including lines [106-121]. Choose an appropriate section title.”

Thank you for this suggestion, which we have incorporated in the revised manuscript.

4. “Before stating which is the best classifier model at line 115, explain in more details the goal of that model:
   4.1) Generally speaking, you built 6 models whose goal is classifying the 50k posts according to the seven hate-speech types, or assigning the ‘no hate speech is present’ label. You should explicit this goal.
   4.2) Clearly explain how the model is used to derive the 7 machine learning algorithms; additionally, it’s not quite understandable what are those 7 machine learning algorithms.
   4.3) Finally, if for each hate-speech type you used a binary classifier telling whether a post does or does not contain a given type of hate speech, how do you label a post as ‘hate-speech not detected’?”

This is an important point, which we have addressed in the paper in lines 135-149 and the SI on pages 9-10. To clarify our methodology, we now use the term “model architecture” to refer to each of the 8 (previously 6, with 2 added per R3’s suggestion) candidate sets of algorithms. Using each model architecture, we implement 7 algorithms, each of which separately classifies whether a post contains or does not contain a given type of hate speech. The 7 algorithms are entirely independent, each of them learning separately from the training data. We then compare the performance of the 8 model architectures across the 7 algorithms to choose the best performing architecture (i.e., BERT-NN). The results reported in the paper use the results from the 7 BERT-NN algorithms. A post is considered to not contain a particular type of hate speech if the machine classifies it as “0” for that type of hate speech, and is considered not to contain any of the 7 types of hate speech if all 7 algorithms classify it as “0”.

5. “Figures:
   5.1) Provide color-blind friendly figures. For example, use different markers, line sizes, grey shades.
   5.2) Provide higher quality images following PLOS ONE’s guidelines.
   5.3) Fig 1, panel B: it needs a better title, like “Total posts containing N hate speech typologies”; add the x-axis label, something like “N Hate Speech Typologies” or “How Many Hate Speech Typologies”
   5.4) Fig 1, panel C: you cannot clearly detect the ‘-‘ sign before the ‘0.1’ at the end of the scale. Besides, the negative values (negative correlation) should be well separated from the positive ones, otherwise the correlation between Gender and Religion (to cite an example) is doubtful. Moreover, what does the dots’ size mean?”

We thank R2 for these helpful suggestions, which we have incorporated in the revised manuscript. With respect to making our figures color-blind-friendly, we tried various changes to Figures 4 and 5, including different line sizes, markers, and gray shades, as suggested by the reviewer. We found that, largely because the lines are close together, these versions were quite difficult to visually parse. We therefore retained the general form of the figures, but changed the color palette to a color-blind-friendly one suggested by Okabe & Ito (2008). With respect to Fig. 1, Panel C, we made several changes to the figure and caption to improve clarity and readability. We welcome any further suggestions on how to improve our figures.

6. “Line 212: specify that you are talking about the fourth subfigure in Fig. 4.”

Thank you for this suggestion, which we have incorporated in the revised manuscript in line 249.

7. “Supporting Information document:
   7.1) Fig 1, panel B: same suggestions as for the main paper
   7.2) Maybe the ‘Machine Learning Methods and Model Selection’ section could benefit from a figure showing how the generic model is used to classify each post with hate-speech types. I did not understand how the ‘weights’ are assigned to perform binary classification (at the end of the first paragraph of this section)
   7.3) The accuracy should be used only when the dataset is balanced, hence Fig. 2 may show the F1 score instead of the accuracy.”

Thank you for these suggestions, which we have incorporated in the revised manuscript. In the Supporting Information, we clarified our description of the machine learning methods on pages 9-10. Most importantly, we clarified that we use 7 separate models, 1 for each type of hate speech, that do not interact or affect each other in any way. The reference to “weights” was an error in the initial submission and has been removed. We have added SI Figure 3 showing the F1 score for each of these models, but nonetheless retained SI Figure 2 comparing accuracy in case some readers find it useful.

8. “if for each hate-speech type you used a binary classifier telling whether a post does or does not contain a given type of hate-speech, how do you label a post as ‘hate-speech not detected’? From what I understand, a post is labelled as ‘hate-speech not detected’ if for all seven binary classifiers that post is classified as ‘hate-speech not detected’, right?”

This is correct. We consider a post as “hate-speech not detected” if each of the 7 models classifies the post as not containing the relevant type of hate speech. These are the ~43 million posts in the first bar of Figure 1B. We have clarified the text on this point in lines 154-155.

9. “Inside the Supporting Information: “[…] whose output is used for 7 separate densely-connected layers […]”, there are 7 separated layers because each one is used to detect a hate speech type, right?”

R2 is correct that each layer is used to detect a single hate speech type. We have clarified this in in lines 135-149 and in the SI on pages 9-10.

**Response to R3’s Comments**

1. “The title is ambitious and the empirical findings do not provide enough evidence to explain the relationship between offline events and online hate (the authors themselves write this is beyond the the scope of the paper). This initial consideration about the title also affects the general narrative of the manuscript.”

We thank R3 for this comment and agree with R3’s perspective. We propose to change the title of the paper to “Measuring Trends in a Wide Scope of Online Hate Speech Following Offline Trigger Events,” which we think focuses more directly on our contribution. For now, we have not changed the name in the documents so as not to cause any technical issues in the editorial process. We welcome further suggestions regarding the most appropriate name for the paper.

2. “The rationale of the paper is not only short but weak. There is not mention to any theoretical background that help to understand the relationship between offline events and online discourses, which in turn would provide a better justification of the RQ's. Which social science theoretical approaches can support the ideas or hypotheses included in this research? Even with a data-driven approach this paper should reflect on this important matter: How can we theoretically model the connections between offline events - media - hate speech - hate crimes? In fact, the authors stressed the importance of what they call "media attention" to understand the connection between the first three elements, but there not a single mention to well-grounded theoretical approaches that already explain part of this process, such as mediatization or, more specifically, the Agenda-Setting hypothesis. If offline events affect the amount of hate speech through the media, some attention should be payed to explain this relationship. Interestingly, the seminal research also suggest that hate speech derives in more hate crimes, which is a offline event again but of a different nature. Probably, including the theoretical understanding of this (recursive) pipeline would be of significant help for the scientific community.”

R3 correctly points out that the framing and lit review of our initial submission was incomplete, particularly with respect to literatures that connect offline events to online hate and that discuss the role of media and elites. We have addressed this important concern by adding a discussion of these literatures to the introduction in lines 42-57. We have also discussed work that links online hate speech to offline hate crimes in lines 40-41 and 407-410. Finally, we have revised the discussion in the conclusions (page 23) to better align with the revised introduction. We think the revised manuscript strikes a better balance between a thorough lit review and our own work, but would be happy to add further to the lit review if the reviewers and editors believe doing so would further improve the paper.

3. “The manual classification for the training corpus (made by 4 coders) should be better explained (in SI or in the paper). First, which kind of training received these coders?, were they supervised (i.e. by choosing and double-checking a random sample of the messages they coded)?; Second, and more important, what is the reliability of the manual annotation?, is there an inter-coder agreement test that guarantees the reliability of the coding process? Were some messages coded simultaneously by 2 or more coders?, and if it was the case, what happened to those messages in which there was not agreement? This may seem a small detail in the method, but I consider that the first step to obtain trust in the ML models is to have a really good training corpus and to be sure the the manual classification was of high quality.”

We thank R3 for raising this important issue, which pointed us to the fact the initial submission included insufficient information regarding our training data. In the revised submission, we added information on this on pages 4-7 of the Supplementary Information. First, we note that two of the coders were subject-matter experts on our team with years of experience studying online hate speech. They trained the other two coders using an extensive set of examples and practice posts. Finally, we conducted an inter-coder reliability exercise in which two of the coders re-coded samples of posts coded by others. This analysis indicates the training data set is highly reliable. We would be glad to provide any additional information regarding our training data that may be necessary.

4. “Regarding the "manual review" of the hate posts, the authors should better explain how it was done (qualitatively, I guess) (and how many of them were reviewed before going to topic modeling) and specially how they linked the topics to the offline events. Did they build a list of offline events? Or how they decided to mention the set offline events included in the manuscript and not some others. Could they bring some agenda-setting studies to get the events with more media attention during the studied period? Moreover, when linking the spikes of hate speech to the events using the main topic detected with LDA, I wonder which other topics were found (I didn't see it in the SI) and if they could be meaningful in some way.”

We have made several revisions to the manuscript and SI to address these important questions. First, we clarified on lines 207-208 and 210-212 that the 4 four spikes in hate posts we analyzed are the 4 largest ones in our time-series. Second, we clarified on lines 218-223 of the paper that, for each of the 4 spikes in hate speech, we reviewed a random sample of 500 posts that contained the applicable type of hate. We did so qualitatively. In each case, it was quite clear which offline event was being discussed in these posts. In other words, the process for choosing the offline events discussed in the manuscript was: (a) quantitative determination of the 4 largest spikes in hate speech; followed by (b) qualitative determination of which key offline events that occurred at that time were discussed in the relevant posts. In future work, we hope to build a more comprehensive set of candidate offline events and analyze their relationship to online hate speech, but that is beyond the scope of this paper.

We think the offline events discussed during these spikes in hate speech are quite clear, but nonetheless we included topic modeling with respect to events 1 and 2 is included simply to validate this qualitative determination. These events may be less well-known to readers as they received much less media attention than the George Floyd death and U.S. Election. Finally, R3 asks about the other topics detected by the LDA for those events. We have not included them in the SI because; (a) they have lower coherence than the topics we did include, and thus might reflect more noise than meaningful discussion – in fact, it was not clear at all that these reflected a coherent substantive topic; and (b) they do not appear related to any specific offline event. We’ve therefore excluded them from the SI because we think they might be more likely to add confusion than clarity, but would be glad to add them going forward if the editors and reviewers request them.

5. “When choosing the ML models, I wonder why specific NN were not tested, even when they are state-of-the-art in supervised text classification. Specifically, I am referring to RNN and CNN, which had been used in many previous studies of hate speech detection.”

We thank R3 for this important suggestion. In performing our revisions, we tested two additional model architectures as R3 suggested: CNN and an implementation of RNN we refer to as Bi-LSTM. We found that the basic neural network we used in the initial analysis out-performed these two alternatives. We discuss this in Section 3 of the Supporting Information.

6. “Some minors details should be addressed such as the correct references to figures in line 266 (p. 17).”

We thank R3 for this pointing out this error, which we have corrected in the revised manuscript in lines 301-303.
